# Tunable deterministic lateral displacement of particles flowing through thermo-responsive hydrogel micropillar arrays

**DOI:** 10.1038/s41598-023-32233-z

**Published:** 2023-03-27

**Authors:** Naotomo Tottori, Takasi Nisisako

**Affiliations:** 1 Department of Mechanical Engineering, School of Engineering, Tokyo Institute of Technology, Tokyo, Japan; 2 Laboratory for Future Interdisciplinary Research of Science and Technology (FIRST), Institute of Innovative Research, Tokyo Institute of Technology, R2-9, 4259 Nagatsuta-cho, Midori-ku, Yokohama, 226-8503 Japan; 3 Present Address: Department of Mechanical Engineering, Faculty of Engineering, Kyushu University, Fukuoka, Japan

**Keywords:** Engineering, Lab-on-a-chip, Gels and hydrogels

## Abstract

Deterministic lateral displacement (DLD) is a promising technology that allows for the continuous and the size-based separation of suspended particles at a high resolution through periodically arrayed micropillars. In conventional DLD, the critical diameter (*D*_c_), which determines the migration mode of a particle of a particular size, is fixed by the device geometry. Here, we propose a novel DLD that uses the pillars of a thermo-responsive hydrogel, poly(*N*-isopropylacrylamide) (PNIPAM) to flexibly tune the *D*_c_ value. Upon heating and cooling, the PNIPAM pillars in the aqueous solution shrink and swell because of their hydrophobic-hydrophilic phase transitions as the temperature varies. Using the PNIPAM pillars confined in a poly(dimethylsiloxane) microchannel, we demonstrate continuous switching of particle (7-μm beads) trajectories (displacement or zigzag mode) by adjusting the *D*_c_ through temperature control of the device on a Peltier element. Further, we perform on/off operation of the particle separation (7-μm and 2-μm beads) by adjusting the *D*_c_ values.

## Introduction

Separation and purification of the suspended particles of interest are crucial for the sample-preparation steps in analytical chemistry. Currently, various microfluidic technologies for particle separation are attracting attention because they can minimize the volumes of precious samples and reagents as well as integrate other elements for comprehensive analysis^1–3^. For example, deterministic lateral displacement (DLD)^4^ is a promising technology that allows for the continuous separation of suspended particles based on their effective sizes at a high resolution through the pillar array^5,6^. To date, the DLD technology has been proven promising for the separation of various particles, including synthetic beads in the nanometer to millimeter range^4,7–9^, water-in-oil and oil-in-water emulsion droplets^10,11^, and biological particles in the nanometer (e.g., DNA^4,12^ and exosomes^7^) to micrometer (e.g., blood cell subtypes^13–16^, circulating tumor cells^17–22^ and viable mammalian cells^23^) range.

A drawback of traditional DLD is that each DLD array can be effective only for a narrow range of predetermined particle sizes. This is because the critical diameter (*D*_c_), which determines the migration of a particle of a particular size through the pillar array, is generally fixed by the device geometry. The fixed *D*_c_ can cause complications when processing biological particles, whose sizes vary considerably by the exact experimental conditions. Also, fouling and non-specific adsorption on the channel surface can unwantedly change the *D*_c_ over time, degrading the separation performance of the device. Thus, there has been a significant demand for methods capable of modulating the *D*_c_ actively to allow using the same DLD device for multiple applications under optimized conditions.

Recently, novel approaches to tune *D*_c_ actively have been developed. One approach is to actively change the device geometry by stretching the elastic device^24^ or changing the flow direction with respect to the pillar array^9,25,26^. Electric field coupling has also been investigated^19,27–30^. Furthermore, streamline evolution and vortex emergence under a high Reynolds number^31,32^, electrostatic effects within the buffer solution at different ionic concentrations^33^, and shear-thinning and elastic effects in non-Newtonian fluids^34^ have been used to actively modulate the *D*_c_ of the device. Thus, methods for the dynamic control of *D*_c_ have been extensively investigated.

**Figure 1:**
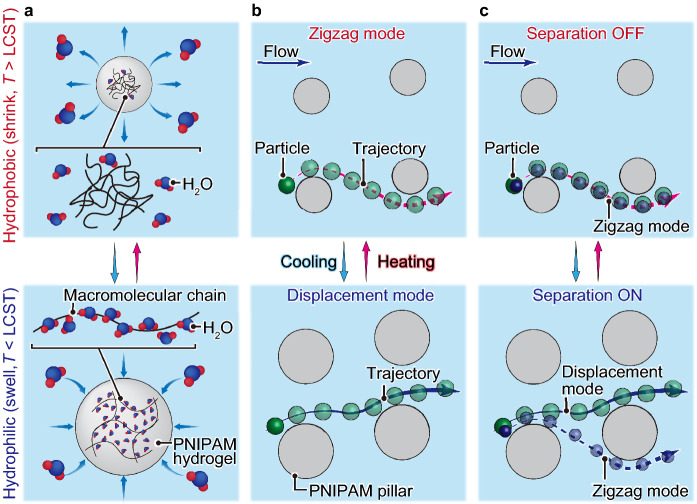
Thermally-tunable deterministic lateral displacement (DLD) of particles flowing through micropillar arrays made of poly(*N*-isopropylacrylamide) (PNIPAM) hydrogel. (**a**) Schematic illustration showing the volume phase transition of PNIPAM hydrogel across lower critical solution temperature (LCST). (**b,c**) Schematic illustration explaining the two experiments demonstrated in this study. (**b**) Thermo-responsive switching of the particle trajectories between the displacement and zigzag modes. (**c**) Thermo-responsive on–off operation in the DLD separation of the two particle populations of different sizes.

Various stimuli-responsive hydrogels have been used as microfluidic components, including actuators, valves, pumps, and particle-trapping structures^35,36^. However, to the best of our knowledge, stimuli-responsive hydrogels have not been applied to DLD. Here, we propose a novel *D*_c_-tunable DLD that uses the pillars of a thermo-responsive hydrogel, poly(N-isopropylacrylamide) (PNIPAM) (Fig. 1). In an aqueous solution, the PNIPAM pillars shrink and swell upon heating and cooling owing to their hydrophobic-hydrophilic phase transition across the lower critical solution temperature (LCST). We perform two experiments to demonstrate the tunability of the *D*_c_ using this thermally tunable device geometry. The first is to switch the trajectories of the single-sized 7-μm particles between the displacement and zigzag modes by thermally shifting *D*_c_ across the particle size. The other is the on–off operation to separate particles of different sizes (7 μm and 2 μm) by similarly shifting the *D*_c_.

## Device design and mechanism

**Figure 2:**
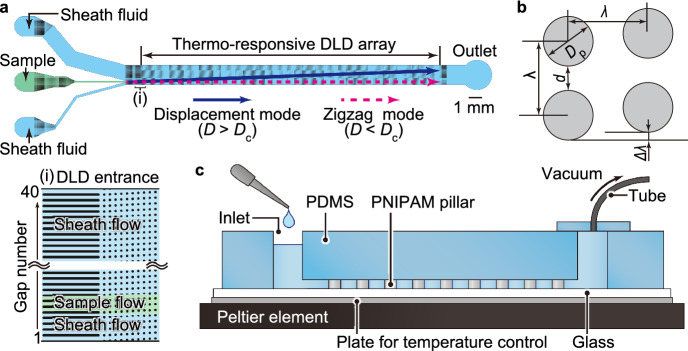
A thermally-tunable microfluidic DLD device. (**a**) Schematic illustration of the thermo-responsive DLD device. The inset illustrates the entrance of the DLD array, and each gap between the pillars is numbered from 1 to 40. (**b**) A rhombic unit cell with the array parameters. *D*_p_ = 20 μm, *λ*  = 50 μm, Δ*λ*/*λ* = 0.05, and *d* = *λ* – *D*_p_ = 30 μm. (**c**) Schematic side view of the device.

We designed a device comprising PNIPAM-based DLD micropillars confined in a poly(dimethylsiloxane) (PDMS) microfluidic channel (Fig. 2a). The PNIPAM-based DLD micropillars in a rhombic cell (Fig. 2b) are arranged in a straight main channel (2 mm wide, 30 mm long, and 10 μm deep) between the three inlets and an outlet. An aqueous solution containing particles is infused in a sheath-focusing regime under negative pressure and the particles start migrating near the right sidewall of the main channel. A Peltier element is placed underneath the device to control the temperature (Fig. 2c).

In the original DLD theory, particles migrate in either of the two modes, displacement or zigzag, as determined by the *D*_c_ defined by the array geometry. Particles larger than *D*_c_ follow the array inclination (displacement mode), while those smaller than *D*_c_ follow the average fluid flow direction (zigzag mode). However, many experiments revealed that a rich class of intermediate migration behavior also exists; their underlying physics is explained by anisotropic permeability^37^ or flow symmetry breaking^38^.

In this study, for simplicity, we approximated the *D*_c_ of our device by using the following empirical formula for binary separation^39^:

1 $${D}_{\mathrm{c}}=1.4\times d\times {(\Delta \lambda /\lambda )}^{0.48}$$

where *d* is the gap size between two adjacent pillars, Δ*λ* is the row-to-row shift of the pillars, and *λ* is the center-to-center distance between the two adjacent pillars (Fig. 2b). Using this empirical equation, we designed a DLD array with a pillar diameter of 20 µm, *d* = 30 µm, and Δ*λ*/*λ* = 0.05, setting a *D*_c_ value of 10 µm under dry conditions. A single DLD section has pillars in 20 columns (= *λ*/Δ*λ*) and 39 rows; 30 such sections are arranged serially such that particles larger than *D*_c_ are sufficiently displaced at the channel end.

The *D*_c_ in our device is thermally tunable via the volumetric phase transition of the PNIPAM micropillars (Fig. 1a). When the device temperature is set below the LCST, the pillars become more hydrophilic and swell, decreasing their gap sizes *d* and *D*_c_. In contrast, when the device temperature is set above the LCST, the pillars become more hydrophobic and shrink, increasing their gap sizes* d* and *D*_c_. Two experiments were conducted to validate this thermally tunable *D*_c_. First, we varied the *D*_c_ of the device by changing the temperature across the LCST to switch the trajectory of single-sized particles (Fig. 1b). Second, we varied the *D*_c_ of the device similarly to turn the separation of the differently sized particles on and off (Fig. 1c).

## Methods

### Fabrication of PNIPAM micropillars

**Figure 3:**
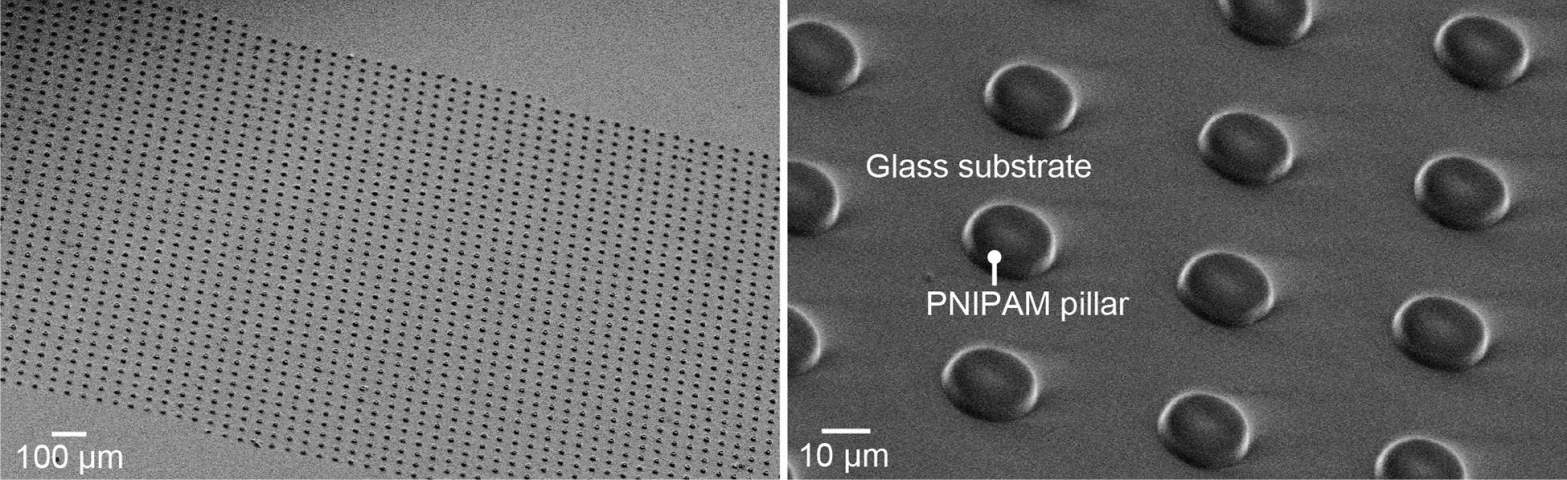
Scanning electron microscopic images of the PNIPAM pillars on a glass slide.

First, a silane coupling agent as an adhesion promoter (Nissan Chemical, Tokyo, Japan) was spin-coated on a glass slide (76 mm × 26 mm, thickness 0.9–1.2 mm) at 2000 rpm for 60 s and the glass slide was baked on a hotplate at 120 °C for 10 min. Subsequently, a PNIPAM-based negative photoresist (Nissan Chemical, Tokyo, Japan) was spin-coated onto a glass slide at 500 rpm for 30 s and baked at 100 °C for 20 min. We repeated the coating and prebaking processes of the photoresist to obtain a final layer thickness of ~ 10 μm. We projected the pattern of micropillars (diameter 20 μm) onto a photoresist-coated glass slide using a laser-printed film mask (thickness of 0.175 mm, resolution of 25,400 dpi; Unno Giken, Tokyo, Japan) and a mask aligner (MA-10, Mikasa, Tokyo, Japan). After being baked at 120 °C for 20 min, the photoresist layer was developed in pure water at room temperature and rinsed in pure water at 60 °C for 20 min. Finally, we baked the glass slide on a hotplate at 60 °C for 20 min to obtain the final PNIPAM micropillars in a dry condition (Fig. 3).

### Fabrication and assembly of the PDMS microchannel

The PDMS component was fabricated using conventional soft lithography. A negative photoresist (SU-8 3025, Nippon Kayaku, Tokyo, Japan) was spin-coated onto a silicon wafer at 5000 rpm to obtain a thickness of ~ 10 µm. After being prebaked on a hotplate at 95 °C for 40 min, the SU8-coated substrate was exposed to ultraviolet light through a laser-printed film mask to obtain the desired channel design. After being baked at 95 °C for 15 min, the photoresist layer was developed using an SU-8 developer to obtain the SU-8 mold. The SU-8 mold and 0.5 mL of chlorotrimethylsilane (Tokyo Chemical Industry, Tokyo, Japan) were confined together in a sealed disposable Petri dish such that the surface of the mold was silanized for easy release of the PDMS replica. The precursor and curative agents of PDMS (Toray, Tokyo, Japan) were mixed at a weight ratio of 10 to 1 and then degassed in a vacuum chamber for 1 h to remove air bubbles. Next, the mixture was cast onto the SU-8 mold, degassed again, and baked on a hotplate at 80 °C for 1 h for curing. Subsequently, the cured PDMS sheet was peeled from the master mold and holes for the inlets and an outlet with a diameter of approximately 2 mm were created using a biopsy punch (Kai Industries, Gifu, Japan). The replicated PDMS microchannel and glass slide with PNIPAM micropillars were manually aligned using an optical microscope and bonded using the self-sticking property of PDMS.

### Sample solutions and sheath fluid

Two aqueous suspensions containing fluorescent polymer microspheres were prepared. One suspension contained fluorescent polymer microspheres with a mean diameter of 7.32 μm (excitation wavelength of 480 nm, emission wavelength of 520 nm, FS06F, Bangs Laboratories, IN, USA). The other suspension contained two populations of fluorescent polymer microspheres with a mean diameter of 7.32 μm and 2.07 μm (excitation wavelength of 360 nm, emission wavelength of 420 nm, FS05F, Bangs Laboratories, IN, USA) with a ratio of 1:1. Both aqueous suspensions were supplemented with 0.1% (v/v) Tween 20 reagent (Sigma-Aldrich, MO, USA) to prevent beads aggregation. The final concentration of the fluorescent beads in the solutions was adjusted to 1.0 × 10^6^ particles/ml. An aqueous solution containing 0.1% (v/v) Tween 20 was prepared as the sheath fluid. Before use, the microchannel was filled with sheath fluid and the prepared bead suspension was injected into the inlet reservoir for the sample and introduced into the microchannel.

### Peripheral equipment and procedure

A gas-tight glass syringe (1000 Series, Hamilton Company, Reno, NV, USA) was connected to the outlet reservoir via a polyethylene tube (inner diameter of 0.5 mm, outer diameter of 1 mm) inserted into the hole of a flat PDMS sheet and mounted on a syringe pump (KDS210, KD Scientific, USA). Next, the sample and sheath fluid solutions were infused into the microchannel by withdrawing the syringe plunger. Particle trajectories through the DLD arrays were observed and recorded using an upright fluorescence microscope (BX51, Olympus, Tokyo, Japan) connected to a digital video camera (HC-750M, Panasonic, Osaka, Japan). The microfluidic device was mounted on an air-cooling Peltier plate (plate size 22 mm × 22 mm, CHP-22HS, Sensor Controls, Kanagawa, Japan) connected to a temperature controller (accuracy ± 0.1 °C, FC-2410, Sensor Controls, Kanagawa, Japan) for temperature control in the microchannel.

### Measurement and image analysis procedure

A superimposed image was generated from the recorded photomicrographs to visualize the trajectories of the fluorescent beads flowing through the DLD arrays. The background image was subtracted from the image of each frame to clarify the bead trajectories. The number of beads passing through each DLD gap was counted from the recorded videos to evaluate the distribution of particles flowing through the DLD array.

## Results and discussion

### Characterization of the PNIPAM micropillars

First, we characterized the shapes of the unconfined PNIPAM pillars under dry conditions. As shown in the scanning electron microscopy images in Fig. 3, we fabricated a periodic array of cylindrical PNIPAM micropillars on a glass slide. The average diameter of the PNIPAM pillars *D*_p_ was 21 μm. Optical measurements validated that the height of the pillars was approximately 10 μm (see Supplementary Fig. S1 online).

Next, we characterized the geometrical parameters of the PNIPAM micropillars in a microchannel filled with an aqueous solution at different temperatures. We introduced aqueous buffer solutions from the inlets to the microchannel by applying a negative pressure at the outlet. Subsequently, we adjusted the device temperature on the Peltier plate and waited for at least a few minutes until the temperature in the microchannel reached the assigned value. Next, we measured the *D*_p_ of the PNIPAM micropillars at a given temperature using optical microscopy.

**Figure 4:**
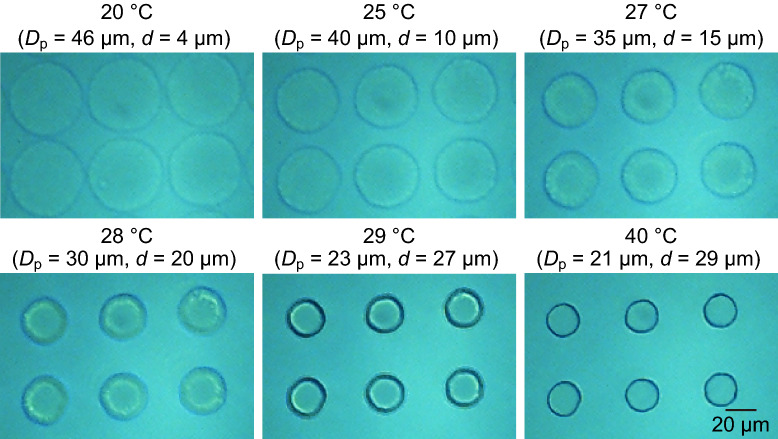
Top-view photomicrographs of the PNIPAM micropillars confined in the PDMS microchannel filled with the aqueous solution at different temperatures.

When we infused the buffer solution and set the device temperature at 20 °C, the average *D*_p_ increased to 46 μm. The gap between the pillars *d* consequently decreased to 4 μm, whereas the pitch between the pillars *λ* maintained a distance under dry conditions (Fig. 4). When the device temperature was gradually increased from 20 to 40 °C, the *D*_p_ decreased and *d* consequently increased; however, *λ* remained unchanged. The *D*_p_ significantly decreased when the device temperature was increasing around 28 °C; the percentage of the change in the *D*_p_ before and after temperature variations at 20–25 °C, 25–27 °C, 27–28 °C, 28–29 °C, and 29–40 °C were 13%, 13%, 14%, 23%, and 9%, respectively. After heating, the substrate was cooled from 40 to 20 °C. In contrast to the heating process, the *D*_p_ increased and *d* consequently decreased, whereas the *λ* remained unchanged. The percentage of the changes in the *D*_p_ before and after temperature variation at 40–29 °C, 29–28 °C, 28–27 °C, 27–25 °C, and 25–20 °C were 9%, 23%, 14%, 13%, and 13%, respectively. Thus, the *D*_p_ of the PNIPAM pillars in the aqueous solution changed reversibly upon heating and cooling. No notable damage was found in the pillars when the heating and cooling cycle was repeated dozens of times.

**Figure 5:**
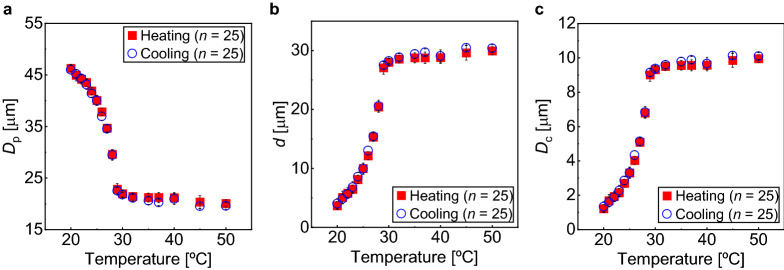
Thermal effects on (**a**) pillar diameters (*D*_p_), (**b**) gap between the two adjacent pillars (*d*), and (**c**) critical diameters (*D*_c_) calculated with Davis’s formula^39^, Eq. (1), of the DLD array. The filled rectangles represent the condition under which the temperature increased from 20 °C to 50 °C. The open circles represent the condition under which the temperature decreased from 50 °C to 20 °C.

We plotted the change in pillar diameter, *D*_p_ and the pillar gap *d* changed with heating and cooling (Fig. 5a,b). When the device was heated from 20 to 25 °C, the *D*_p_ gradually decreased from 46 to 40 μm (at the rate of −1.2 μm/℃), increasing the *d* from 4 to 10 μm. From 25 to 29 °C, the decrease in the *D*_p_ was more severe from 40 to 23 μm (−4.3 μm/℃), increasing the *d* from 10 to 27 μm. Above 30 °C, the *D*_p_ was almost maintained at 21 μm (*d* = 29 μm) (Fig. 5a,b). Subsequently, the device was cooled. The *D*_p_ was almost maintained at 21 μm down to 30 °C. From 29 to 25 °C, the *D*_p_ significantly increased from 23 to 40 μm, decreasing the *d* from 27 to 10 μm. From 25 to 20 °C, the *D*_p_ gradually increased from 40 to 46 μm, decreasing the *d* from 10 to 4 μm. Thus, we validated the characteristic volume shift of the PNIPAM pillars with negligible hysteresis when the device was heated and cooled across the transition temperature.

Furthermore, we calculated the *D*_c_ values by substituting the measured *d* values at each temperature and setting Δ*λ*/*λ* (= 0.05) in Davis's formula^39^, Eq. (1) (Fig. 5c). When the device was heated from 20 to 25 °C, for example, the *D*_c_ gradually increased from 1 to 3 μm. In contrast, the *D*_c_ significantly changed from 3 to 9 μm from 25 to 29 °C. Above 30 °C, the *D*_c_ was almost maintained at 10 μm. Upon cooling, the *D*_c_ changed reversibly without significant hysteresis. Consequently, the variable range of *D*_c_ was between 1 and 10 μm, indicating that the maximum change rate of *D*_c_ was approximately ten folds.

We evaluated the effect of height restriction on the *D*_p_ by comparing the confined and unconfined PNIPAM pillars. When the unconfined PNIPAM pillars in the aqueous solution were heated and cooled, the *D*_p_ changed reversibly between 45 and 20 μm (see Supplementary Fig. S2 online). The *D*_p_ of the confined and unconfined PNIPAM micropillars at each temperature showed almost no difference (see Supplementary Fig. S3 online). However, at a low temperature of approximately 20 °C, the *D*_p_ of the unconfined PNIPAM micropillars was slightly lower than that of the confined pillars. The unconfined PNIPAM pillars should transform isotropically^35^. In contrast, we consider that the height restriction by confinement can result in a slight horizontal expansion of the PNIPAM pillars swelling at a low temperature. Also, the bonding between the PNIPAM pillars and the glass surface might be forming the non-cylindrical pillars upon swelling, although it was unclear from the top view.

**Figure 6:**
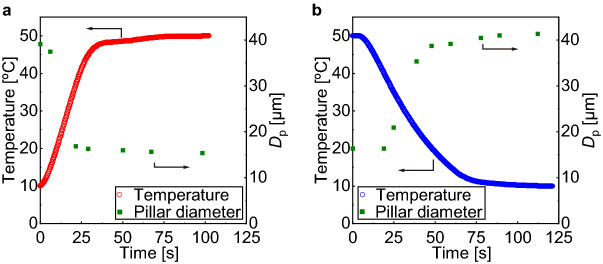
Response time measurement of the temperature of the Peltier plate and the pillar diameters. The open circles represent the temperatures recorded over time after the preset temperature was abruptly changed (**a**) from 10 °C to 50 °C and (**b**) from 50 °C to 10 °C at *t* = 0. The solid rectangles represent the variation of the pillar diameters in response to the temperature change.

Further, we evaluated the response speed of the volumetric shift of the PNIPAM pillars upon temperature variation by the Peltier plate. When the set temperatures of the Peltier plate changed from 10 to 50 °C and from 50 to 10 °C abruptly, the time constant (*τ*) was 20 s (corresponding to 35 °C) and 40 s (corresponding to 25 °C), respectively (Fig. 6). The change in *D*_p_ smoothly followed this variation in device temperature (Fig. 6, see Supplementary videos online). The *D*_p_ varied from the maximum to minimum diameter within 21 s and from minimum to a maximum diameter within 59 s when the temperature of the Peltier plate increased from 10 to 50 °C and decreased from 50 to 10 °C, respectively. From the elapsed time for sufficient shrinking and swelling, we roughly estimated that the solution’s incoming and outgoing averaged heat flux was 80.0 and 28.5 W/m^2^, respectively.

### Switching the trajectories of the particles through the PNIPAM micropillars

We used a suspension of single-sized (7 μm) fluorescent particles to demonstrate their thermally switchable trajectories through PNIPAM pillars. First, the buffer solution (tens of microliters) was injected into the three open-inlet reservoirs and later withdrawn into the channel under negative pressure. After the entire channel was filled with the buffer solution, the solution in the middle inlet reservoir was replaced with a suspension of 7-μm beads (tens of microliters). The suspension was withdrawn into the channel along with sheath fluid.

**Figure 7:**
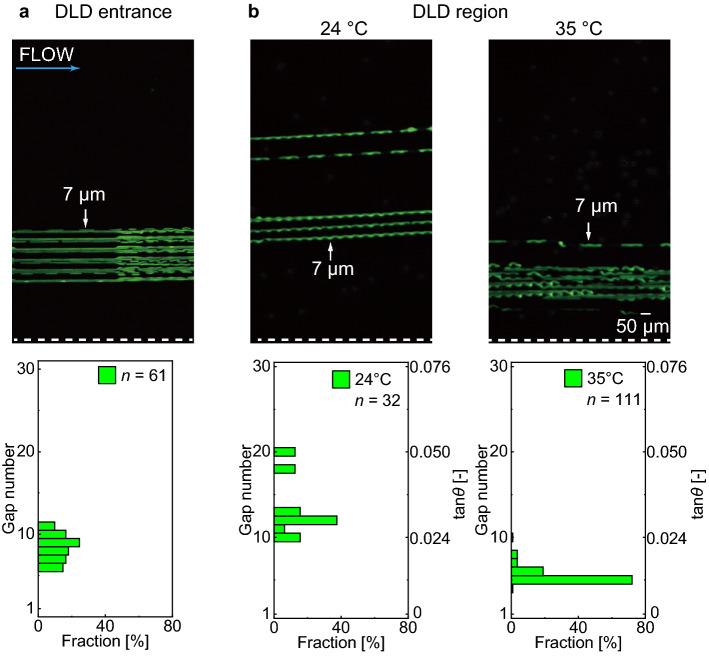
Thermally-switchable particle trajectories. (**a**) Trajectories and spatial distributions of the 7-μm beads (green) in the focused stream at the DLD entrance. (**b**) Trajectories and spatial distributions of the 7-μm beads flowing downstream (20th DLD section) in displacement mode at low temperature (24 °C, left) and in zigzag mode at high temperature (35 °C, right).

A fluorescence microscopy image at the entrance of the DLD region when the withdrawing flow rate was set at 0.5 mL/h is shown in Fig. 7a. Thus, we could achieve sheath-focusing of the stream of the particle suspension near the right-side wall with respect to the flow direction at the DLD entrance (GN = 6–11).

When the device temperature was set at 24 °C, well below the LCST, the 7-μm beads in the sheath-focused stream migrated toward the left-side wall with respect to the flow direction in displacement mode (Fig. 7b, left). This migration mode properly agrees with the fact that the calculated *D*_c_ at 24 °C was ~ 3 μm (Fig. 5c), which was smaller than the diameter of the 7-μm beads. The measured mean velocity of the 7-μm beads flowing through the DLD array was 3.1 ± 0.1 mm/s (*n* = 11). From the mean velocity of the particles (*v* = 3.1 mm/s), fluid density (*ρ* = 998.2 kg/m^3^), gap between the posts (*d* ~ 10 μm), and fluid viscosity (*μ* = 1.002 mPa s), the Reynolds number (Re = *ρvd*/*μ*) was calculated to be O(−2), indicating a sufficiently laminar flow through the DLD array.

In contrast, when the device temperature was set at 35 °C, well above the LCST, the 7-μm beads flowed in the zigzag mode (Fig. 7b, right), maintaining approximately the initial gap positions (GN = 4–10). This migration mode also agrees with the calculated *D*_c_ at 35 °C was ~ 10 μm (Fig. 5c), which was larger than the diameter of the 7-μm beads. Under this condition, the measured mean velocity of the beads was 6.0 ± 0.5 mm/s (*n* = 11). This velocity increase from 24 to 35 °C was presumably caused by the decrease in hydrodynamic resistance through the wider gaps in the negative-pressure-driven flow. Thus, we could reproducibly switch the trajectories of the particles between the displacement and zigzag modes by changing the device temperature across the LCST.

### On/off operation of particle separation through the PNIPAM micropillars

We used a suspension of fluorescent particles of two sizes (7 μm and 2 μm) to demonstrate the on/off operation of their separation by modulating the device temperatures. Similar to the experiment with monosized beads, the entire channel was first filled with the buffer solution. Next, we replaced the buffer solution in the middle inlet with the mixture suspension and started its infusion into the channel by applying negative pressure at the outlet.

**Figure 8:**
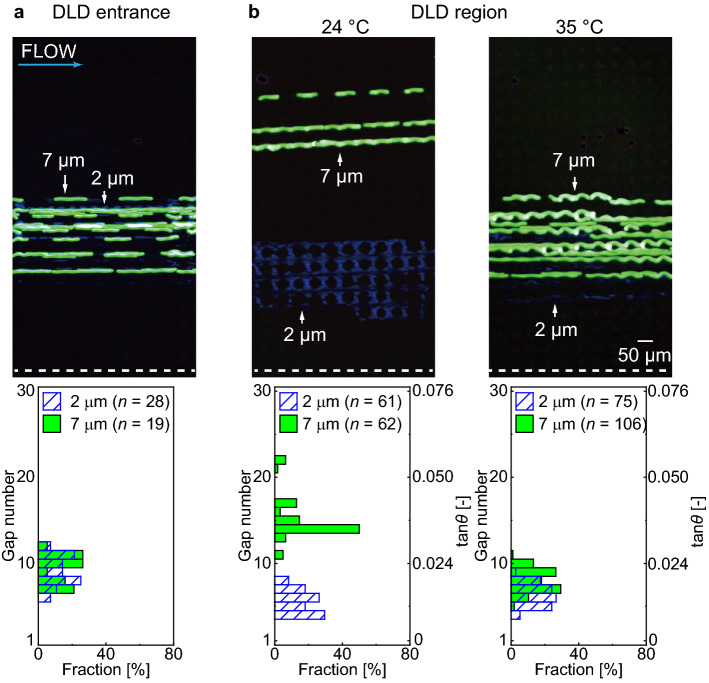
Thermo-responsive on–off operation in the DLD separation of the particles. (**a,b**) Trajectories and spatial distribution of the 7-μm (green) and 2-μm (blue) beads at the DLD entrance. (**b**) Trajectories and spatial distributions of the beads flowing downstream (20th DLD section) at two different temperatures. (left) Separation is ON at a low temperature (24 °C); the 7-μm beads (green) are flowing in displacement mode, and the 2-μm beads (blue) are flowing in zigzag mode. (right) Separation is OFF at a high temperature (35 °C); the 7-μm and 2-μm beads flow in zigzag mode.

A fluorescence microscopy image at the DLD entrance when the withdrawal flow rate was at 0.5 mL/h is shown in Fig. 8a. From the overlaid image, we validated that both 7-μm and 2-μm beads flowed in a focused stream near the right-side wall with respect to the flow direction (GN = 6–12).

An overlaid fluorescence microscopy image of the particles flowing through the micropillar array when the device temperature was 24 °C is shown in Fig. 8b (left). The mean velocity of the particles was 2.2 ± 0.3 mm/s (*n* = 11). Here, the 7-μm beads flowed toward the left-side wall with respect to the fluid direction in the displacement mode (GN = 11–22), whereas the 2-μm beads flowed in the zigzag mode (GN = 4–8). That is, the separation of the 7-μm and 2-μm particles was “ON” when the device temperature was set below the LCST. These two migration modes agree with the fact that the *D*_c_ of the device at 24 °C was ~ 3 μm, which was between the 2-μm and 7-μm particles.

In contrast, when the device temperature was increased to 35 °C, the pillars shrank and the gap increased. Consequently, the 2-μm and 7-μm beads both migrated similarly in the zigzag mode, maintaining approximately the initial vertical positions (GN = 4–11) with respect to the direction of the global fluid stream (Fig. 8b, right). The mean velocity of the particles was 3.3 ± 0.4 mm/s (*n* = 11). Thus, their separation was “OFF” when the device temperature was set above the LCST. This flow behavior agrees with the fact that the *D*_c_ of the device at 35 °C was ~ 10 μm, which was larger than both particles. Thus, we could reproduce the on/off operation of particle separation by switching the device temperatures across the LCST.

The proposed thermally tunable DLD has several advantages over others. Unlike the DLD tuned by stretching^24^, the entire device does not need to be elastic. Unlike devices with a tunable angle of the pillar arrays^9,25,26^, the operation of our device does not need to be suspended to change the device geometry. Compared with electrically assisted DLDs^19,27–30^, our device has no electrodes and can be replicated easily at a low cost via soft lithography. In addition, our device does not rely on high Re^31,32^ and the use of a viscoelastic fluid^34^. Finally, the hydrodynamic resistance of the proposed device will decrease dramatically as the gap size increases upon heating (see Supplementary Fig. S4 online), which will be helpful to flush the device for reuse.

Meanwhile, in a thermally tunable DLD, the heat transfer effect between the supplied solutions and the device must be carefully considered. It is favorable for stable operation provided the initial temperature of the solution is close to the device temperature. Otherwise, the flow rate must be sufficiently low such that the device temperature setting becomes more dominant in tuning the pillar diameters than the incoming solution temperature. Notably, our device consumes a longer time to reach thermal equilibrium when the temperature settings are changed. Additionally, when bioparticles are used, the device temperature should be moderate to avoid damaging the particles.

Upon the phase transition of the PNIPAM pillars, the electrostatic or hydrophilic-hydrophobic interactions between the pillars and particles might be considered. However, these effects were not observed in the experiments. For a detailed evaluation of the pillars (e.g., a cyclic fatigue test), analysis of their 3D shape at various solution temperatures (e.g., by fluorescence-based profilometry^40^) will be necessary. In practical applications, the effect of the solution characteristics, including pH, surfactants, and dissolved salts, on the LCST and swelling behavior of PNIPAM need to be considered^35^. For example, the kinetics of PNIPAM can be improved by numerous physical and chemical strategies^41^.

To enhance the functionality of the device, multistep DLD arrays, having different *D*_c_ in series, could be achieved by applying different temperatures to each section of the micropillar array, enabling the fractionation of particles of various sizes. Although we used only negative pressure to generate fluid flow in the experiments, positive pressure should yield the same results because, in laminar flow, the time reversal does not change the flow trace and will not modify the particle trajectories. For the fluid infusion by positive pressure, a procedure for error-free alignment between the PNIPAM pillars and PDMS channel is needed. Furthermore, other stimuli-responsive hydrogels (e.g., optically responsive and pH-responsive hydrogels) might be applied to fabricate a tunable DLD array. Combined with the precise and strategic device temperature control, these will increase the scope of potential applications, such as in separating and purifying various particles of more than two sizes.

## Conclusion

We proposed a novel DLD device with a thermally tunable *D*_c_ and demonstrated the switching of the particle trajectories and the on/off operation of particle separation. We fabricated DLD pillars using a PNIPAM-based photoresist in a PDMS microchannel to enable the thermal tunability of the *D*_c_ of the DLD array. The trajectories of the 7-μm beads could be switched between the displacement and zigzag modes by continuously changing the *D*_c_ of the array by adjusting the temperatures. In addition, the on/off operation to separate the 7-μm and 2-μm beads could be achieved by adjusting the temperature similarly. Microfluidic devices with thermally tunable DLD arrays have various potential applications, such as in the purification and separation of many types of biological particles, offering the advantages of ease of operation and particle separation capability of various sizes in a single device.

## Supplementary Information

Supplementary Information.

Supplementary Video 1.

Supplementary Video 2.

## Data Availability

The datasets generated during and/or analysed during the current study are available from the corresponding author on reasonable request.
