# Supplementary material for: Tunable deterministic lateral displacement of particles flowing through thermo-responsive hydrogel micropillar arrays
Source: Sci Rep. 2023 Mar 27;13:4994. doi: 10.1038/s41598-023-32233-z (PMC10043002; doi:10.1038/s41598-023-32233-z)
Supplement: Supplementary file 1 — Supplementary Information. [file 41598_2023_32233_MOESM1_ESM.docx]

Tunable Deterministic Lateral Displacement of Particles Flowing through Thermo-Responsive Hydrogel Micropillar Arrays

Naotomo Tottori^1†^ & Takasi Nisisako^2^*

^1^Department of Mechanical Engineering, School of Engineering, Tokyo Institute of Technology, Tokyo, Japan.

^2^Laboratory for Future Interdisciplinary Research of Science and Technology (FIRST), Institute of Innovative Research, Tokyo Institute of Technology, R2-9, 4259 Nagatsuta-cho, Midori-ku, Yokohama, 226-8503, Japan.

^†^Present address: Department of Mechanical Engineering, Faculty of Engineering, Kyushu University, Fukuoka, Japan.

*nisisako.t.aa@m.titech.ac.jp

**Supplementary Information**

**Supplementary Figures**


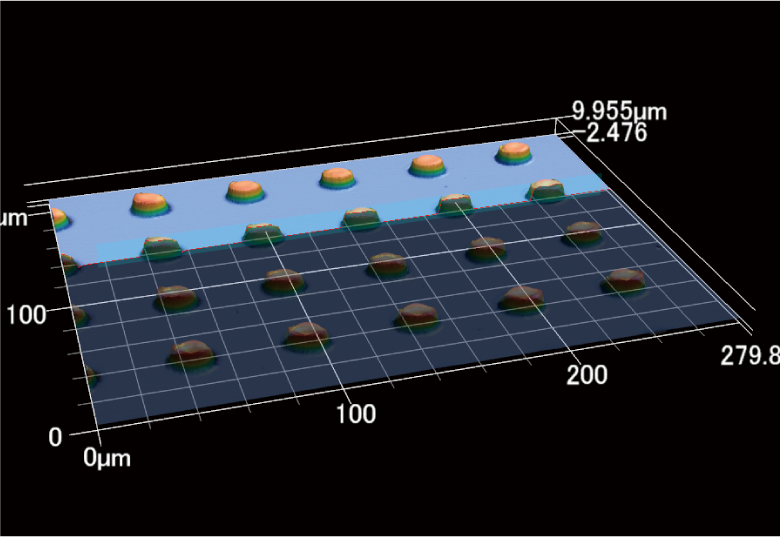


**Figure S1.** Optically measured three-dimensional shapes of poly(N-isopropyl acrylamide) (PNIPAM) micropillars in a dry condition.


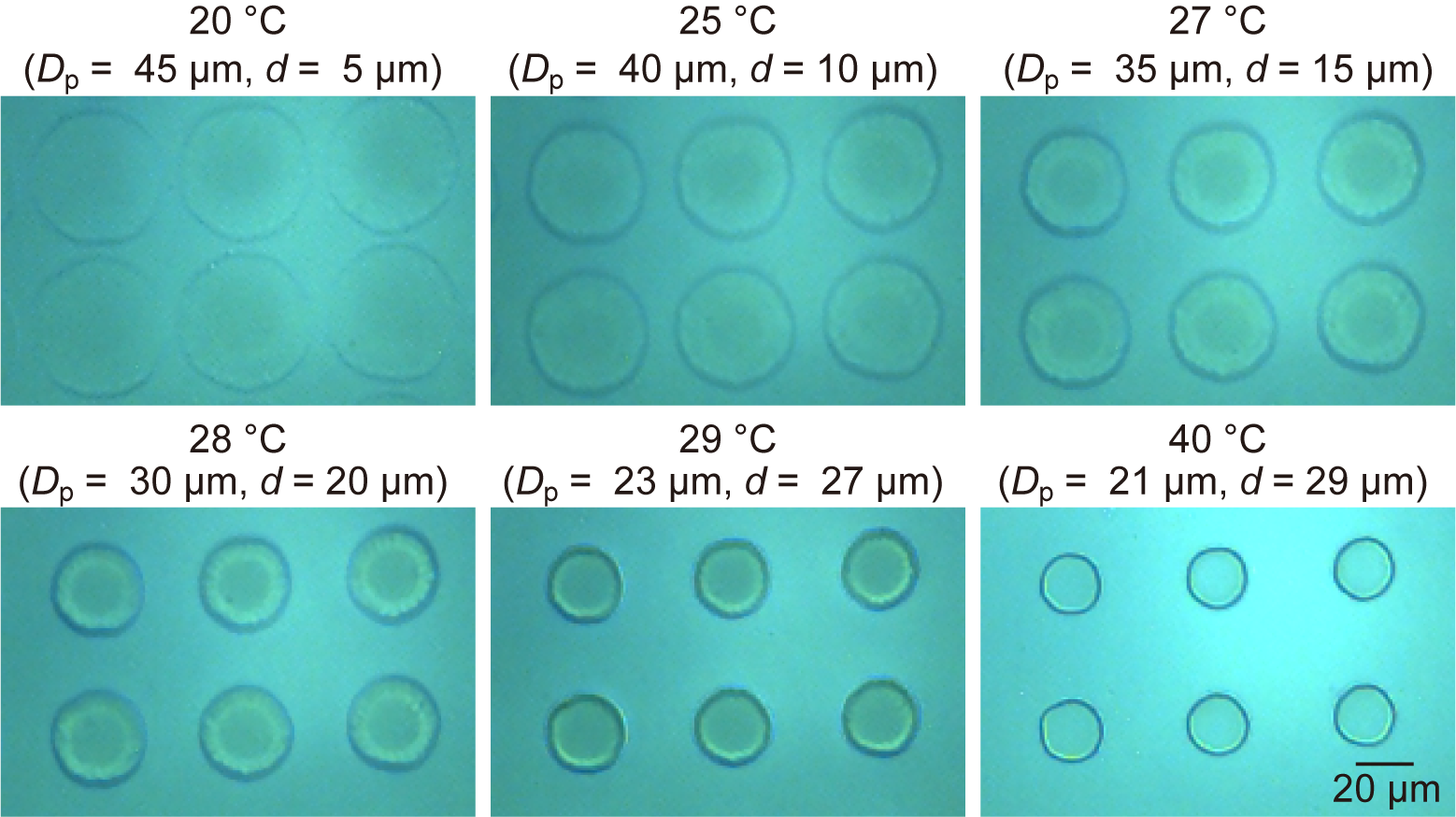


**Figure S2.** Top-view photomicrographs of the PNIPAM micropillars in an aqueous solution at different temperatures without a PDMS channel.


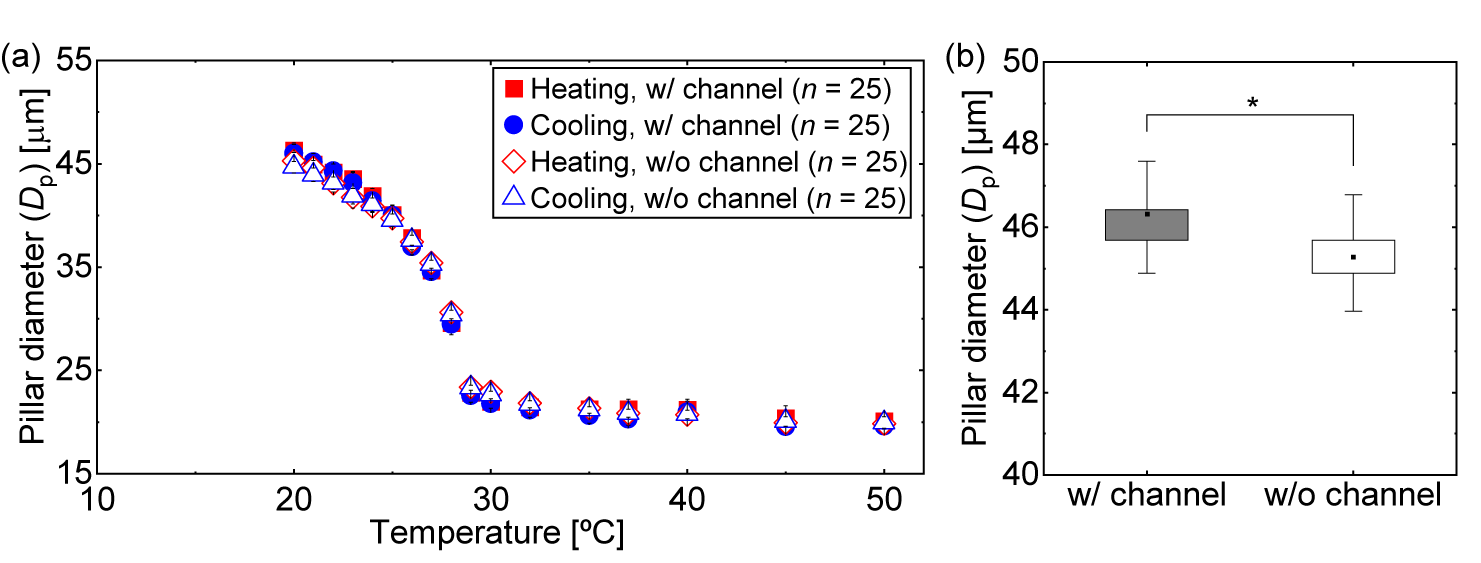


**Figure S3.** Thermal effects on the diameters of the confined and unconfined PNIPAM micropillars. (a) Variation of the diameters of the confined and unconfined PNIPAM micropillars when the device temperature was varied between 20°C and 50°C. (b) Box plot of the diameters of the confined and unconfined micropillars at 20°C for independent samples t-test. The asterisk (*) means a p-value of less than 0.05, indicating a statistically significant difference between the two data.

**Figure S4.** Estimated relation between the volumetric flow rate and device temperature when *ΔP* at 1 atm is applied across the 30 DLD sections (20 × 39 pillars per section). ANSYS Fluent was used for 3D simulation.

**Supplementary Movie Captions**

**Video S1**, Shrinking_by_heating.mov: A real-time movie clip of the PNIPAM-based DLD micropillars shrinking upon heating.

**Video S2**, Swelling_by_cooling.mov: A real-time movie clip of the PNIPAM-based DLD micropillars swelling upon cooling.
